# Repeatability of regional myocardial blood flow calculation in ^82^Rb PET imaging

**DOI:** 10.1186/1756-6649-9-2

**Published:** 2009-01-29

**Authors:** Karin Knešaurek, Josef Machac, Zhuangyu Zhang

**Affiliations:** 1 Division of Nuclear Medicine, The Mount Sinai Medical Centre, New York, NY, USA

## Abstract

**Background:**

We evaluated the repeatability of the calculation of myocardial blood flow (MBF) at rest and pharmacological stress, and calculated the coronary flow reserve (CFR) utilizing ^82^Rb PET imaging. The aim of the research was to prove high repeatability for global MBF and CFR values and good repeatability for regional MBF and CFR values. The results will have significant impact on cardiac PET imaging in terms of making it more affordable and increasing its use.

**Methods:**

12 normal volunteers were imaged at rest and during pharmacological stress, with 2220 MBq of ^82^Rb each. A GE Advance PET system was used to acquire dynamic 50-frame studies. MBF was calculated with a 2-compartmental model using a modified PMOD program (PMOD; University Hospital Zurich, Zurich, Switzerland). Two differential equations, describing a 2-compartmental model, were solved by numerical integration and using Levenberg-Marquardt's method for fitting data. The PMOD program defines 16 standard segments and calculates myocardial flow for each segment, as well as average septal, anterior, lateral, inferior and global flow. Repeatability was evaluated according to the method of Bland and Altman.

**Results:**

Global rest and stress MBF, as well as global CFR, showed very good repeatability. No significant differences were found between the paired resting global MBF (0.63 ± 0.13 vs. 0.64 ± 0.13 mL/min/g; mean difference, -1.0% ± 2.6%) and the stress global MBF (1.37 ± 0.23 vs. 1.37 ± 0.24; mean difference, 0.1% ± 2.3%). Global CFR was highly reproducible (2.25 ± 0.56 vs. 2.22 ± 0.54, *P *= not statistically significant; mean difference, 1.3% ± 14.3%). Repeatability coefficients for global rest MBF were 0.033 (5.2%) and stress MBF 0.062 (4.5%) mL/min/g. Regional rest and stress MBF and CFR have shown good reproducibility. The average per sector repeatability coefficients for rest MBF were 0.056 (8.5%) and stress MBF 0.089 (6.3%) mL/min/g, and average repeatability coefficient for CFR was 0.25 (10.6%).

**Conclusion:**

The results of the study show that software calculation of MBF and CFR with ^82^Rb myocardial PET imaging is highly repeatable for global values and has good repeatability for regional values.

## Background

In noninvasive evaluation of coronary heart disease, cardiac positron emission tomography (PET) has been shown to have high sensitivity and specificity for assessing myocardial perfusion and metabolism [1]. In comparison to single photon emission tomography (SPECT), PET provides accurate nonuniform attenuation correction which allows quantification of various physiologic parameters. PET imaging has the ability to provide noninvasive regional absolute quantification of myocardial blood flow (MBF) and the assessment of coronary flow reserve (CFR). CFR is the ratio of MBF during maximal coronary vasodilatation to resting MBF and has been proposed as an indirect parameter for evaluation of the function of the coronary circulation. Recently, Kaufmann and Camici [2] described the technical aspects and clinical applications of MBF measurement by PET.

The three most widely used PET perfusion tracers are ^13^NH_3_, ^15^O-labeled water (H_2_^15^O), and the cationic potassium analog ^82^Rb [3-9]. Among these tracers only ^82^Rb is generator-produced and does not require an on site cyclotron. The use of ^82^Rb for PET myocardial perfusion imaging is expected to increase in the near future due to widespread availability of this tracer and the dramatic increase in the number of PET scanners that has occurred over the last 10 years. However, there are several issues related to quantification of regional MBF using ^82^Rb. First, cardiac images obtained with ^82^Rb tend to be count-poor due to the short half-life of ^82^Rb (75 s). Second, the high positron energy (3.15 MeV) results in decreased resolution compared to other PET tracers. Third, there is heavy dependence of myocardial extraction of this tracer on the prevailing flow rate and myocardial metabolic state [2].

The low-count imaging has recently been addressed with the higher sensitivity 3D mode of PET imaging evaluated for myocardial perfusion ^82^Rb PET imaging [10,11]. Imaging in the 3D mode is expected to have a higher sensitivity as opposed to 2D imaging, although at the price of high random events and scatter.

In this paper, we wish to evaluate the repeatability of the PMOD software approach for MBF measurements at rest and pharmacological stress, and calculation of CFR utilizing 2D ^82^Rb PET imaging.

## Methods

A GE ADVANCE (General Electric Medical Systems, Milwaukee, WI) system was used for all acquisitions. The system has 18 detector rings and 12,096 bismuth germanate (BGO) 4 × 8 × 30 mm crystals. In the 2D acquisition mode, which was used in this study, the system uses a tungsten collimator 1 × 120 mm in size. The axial field of view is 15.2 cm covered by 35 image planes. The axial sampling interval is 4.25 mm. The transaxial field of view is 55.0 cm. The coincidence window width is 12.5 ns and the energy window is in a range of 300–650 keV. All 2D acquisitions were performed in high sensitivity mode. The images were reconstructed using a filtered backprojection reconstruction method and a Hanning smoothing filter with a 0.5 cy/cm cutoff. The matrix size was 128 × 128 and the pixel size was 4.29 mm. Attenuation correction using an 8-min transmission scan was applied in all studies. In addition, standard corrections for randoms and scatter provided by the vendor were applied. 12 normal volunteers, mean age 35 ± 9.5, were imaged at rest and pharmacological stress, following an i.v. injection of 2220 MBq of ^82^Rb each. Pharmacologic stress was achieved with a standard dose of adenosine (140 mg/kg/min infused over 6 min) or dipyridamole (0.56 mg/kg infused over 4 min). For each dynamic study, 50 frames were acquired. The time per frame was 5 sec between 0–3 min, 15 sec between 3–5 min and 30 sec between 5–8 min. The institutional review board granted ethical approval for the study and each subject signed a consent form.

**Figure 1:**
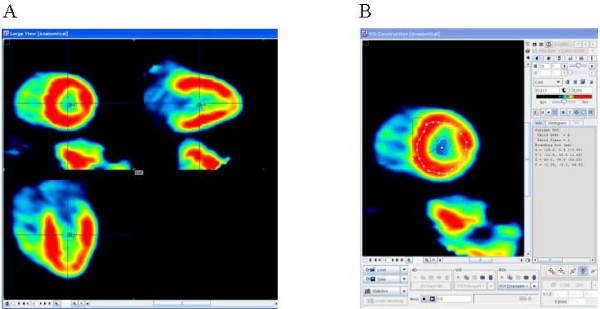
**Short-axis slices**. A) Summed dynamic images were used to reorient images and obtain short-axis slices which B) were used to determine LV ROI and LV blood pool ROI. The ROIs were then used in the original dynamic study in order to create time-activity-curves (TAC).

**Figure 2:**
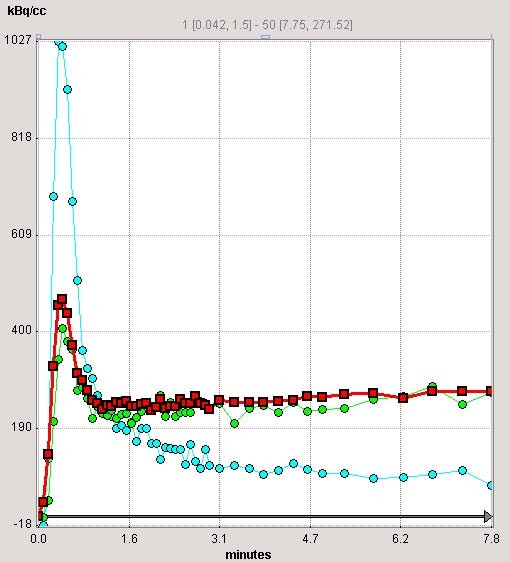
**Time activity curves (TACs)**. PMOD (2.65) creates TACs from different areas of the myocardium as well as from the LV cavity (light blue) which is used as an input curve in the kinetic modelling. In total, 16 LV segments TAC are created in addition to global myocardial curve (shown in red). The apical-anterior segment TAC is shown in light green. The TAC derived from the RV VOI (not shown here) was used for blood spillover correction during kinetic modelling.

**Figure 3:**
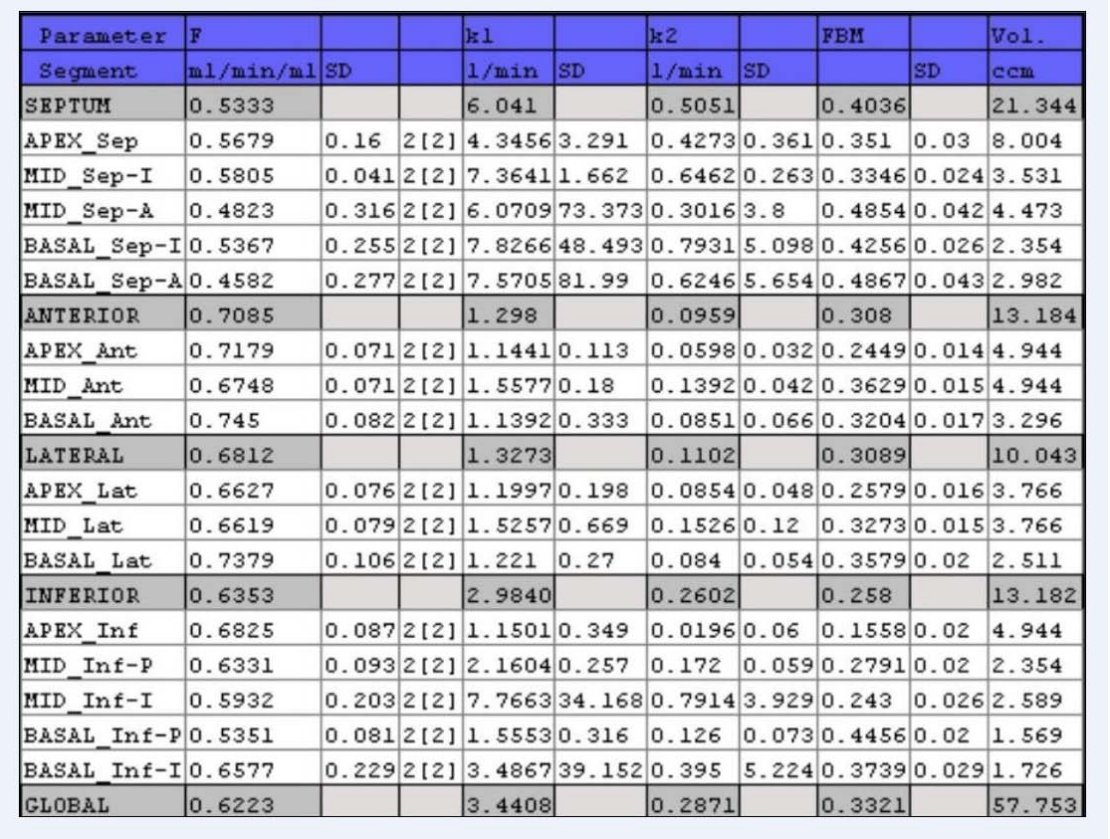
**Results of MBF calculations**. Result of the PMOD (version 2.65) ^82^Rb model program. In addition to flow, it calculates 2-compartmental constants, k1, k2, cross-talk from blood pool to tissue (FBM) and respective standard deviations for 16 standard segments as well as average septal, anterior, lateral, inferior and global area.

In the spring of 2004, we collaborated with PMOD developers from University Hospital of Zurich, Switzerland [12,13], to develop an ^82^Rb model for regional MBF calculation. The PMOD software is developed primarily for image quantification and kinetic modelling of PET data. PMOD has been written in Java 2 and is currently offered under Windows, Linux and MacOS X. As data can be read and written in DICOM, as well as some other formats (GE PET format, Advance, etc.), it can be applied on different PET data and on different computer platforms. We tested a beta version of the PMOD ^82^Rb model, which we used to compare with our results. Our results were obtained using Herrero's 2-compartmental model [6,7] and SAS (SAS Institute Inc., NC) software. The PMOD program had been used before for the assessment of MBF with rest and stress in ^15^O-labeled water PET studies [13], showing good repeatability in rest, adenosine stress and exercise stress studies. The PMOD (PMOD version 2.65) program reads the original data set twice. First, frames are summed in time and used to reorient images from transaxial to short-axis slices and to define volume-of interests (VOIs) (Fig. 1A). From the short-axis slices, the PMOD program defines VOIs over the right ventricle (RV), LV blood pool and over LV myocardium. The junctions of right and left ventricle were marked to indicate the septum (Fig. 1B). In the second reading the dynamic image data are not summed but are temporally smoothed by Daubechies wavelets [14], with the four filter coefficients. The Daubechies wavelets are a family of orthogonal wavelets which are easy to put into practice using the fast wavelet transform and therefore they have been widely used in solving a broad range of problems. The defined VOIs from the first reading are applied to the reoriented original dynamic study, creating input blood pool and myocardial LV segment time-activity-curves (TAC) (Fig. 2). The TAC derived from the RV VOI is used for blood spillover correction during kinetic modelling. The PMOD program defines 16 standard segments [15] and calculates myocardial flow for each segment, as well as average septal, anterior, lateral, inferior and global flow (Fig. 3). The PMOD calculates the MBF using a 2-compartmental model [6-8]. The recovery coefficient (FMM) was set to 0.65, and the fractional volume of the first compartment (V_d_) was fixed at 0.75 mL/mL [6]. Two differential equations, describing a 2-compartmental model, were solved by numerical integration and using Levenberg-Marquardt's method for fitting data. The program calculates flow values (ml/min/ml), k1 and k2 constants (1/min) and cross-talk from blood to tissue (FMB). The PMOD also gives the volume (ccm) for each segment (Fig. 3). The same set of images was analyzed twice in order to investigate repeatability of the analysis.

The Passing & Bablok regression scatter diagrams [16] with the regression line (solid line), the confidence interval for the regression line (dashed lines) and identity line (x = y, dotted line), were used to show rest and stress data for two different runs. The Bland and Altman method [17] was used to analyze the difference between the two measurements and to test the repeatability of each measurement. The repeatability coefficient was calculated as 1.96 times the SD of the differences [18]. The data are reported as mean ± SD. For comparison, the repeatability coefficient is also given as a percentage of the average value of the 2 measurements.

## Results

**Figure 4:**
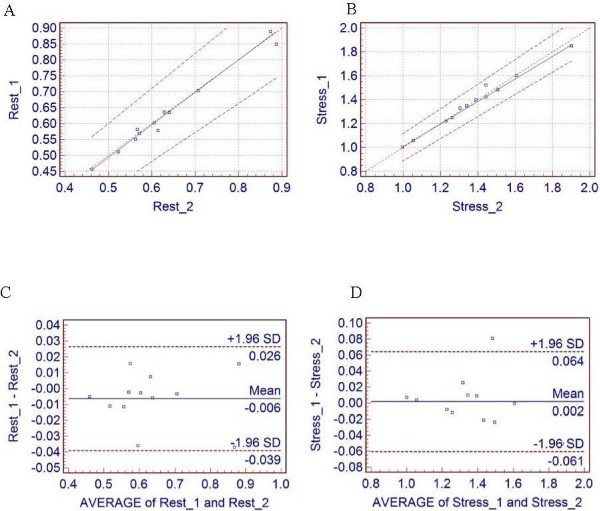
**Global flow results**. The Passing & Bablok regression scatter diagram with the regression line (solid line), the confidence interval for the regression line (dashed lines) and identity line (x = y, dotted line), for global MBF values at (A) rest (n = 12, r = 0.99) and (B) stress (n = 12, r = 0.99) for two different runs. Altman-Bland plots for rest (C) and stress (D) global myocardial blood flow, respectively.

**Figure 5:**
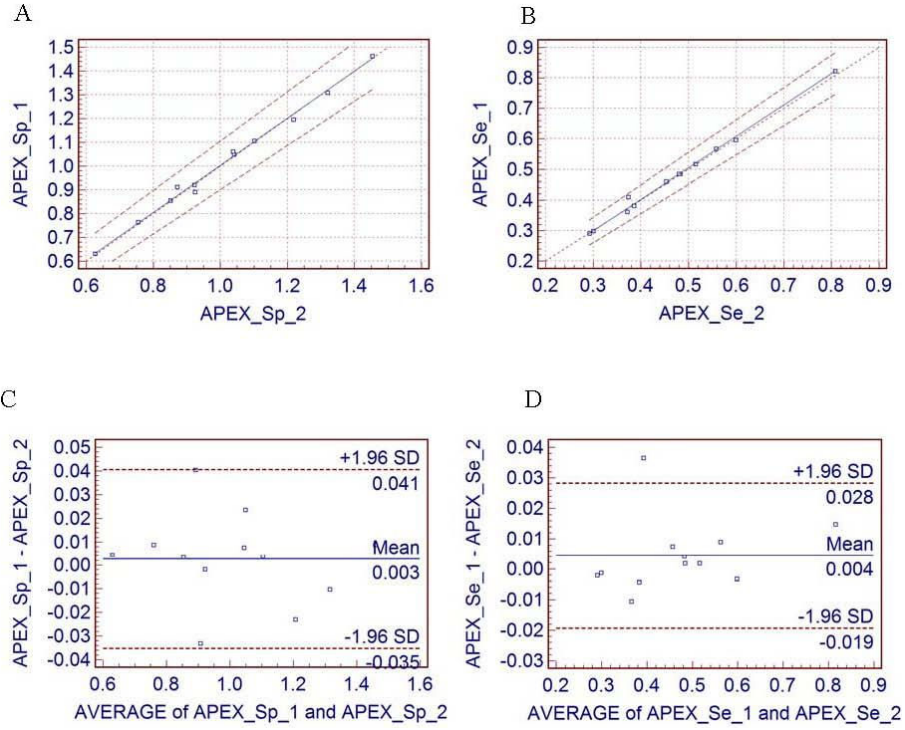
**Regional flow results**. The Passing & Bablok regression scatter diagram with the regression line (solid line), the confidence interval for the regression line (dashed lines) and identity line (x = y, dotted line), for regional apex-septal MBF, at (A) rest (n = 12, r = 0.99) and (B) stress (n = 12, r = 0.99) for two different runs. Altman-Bland plots for the same regional apex-septal MBF, for rest (C) and stress (D), respectively short-axis slices profile ROI.

The average of all 12 subjects, regional and global MBF values at rest and stress are given in Table 1 and Table 2, with corresponding reproducibility and reproducibility expressed as percentage of the average value. Also, the Passing & Bablok regression scatter diagram and the results of the Bland and Altman comparison between two runs of calculating rest and stress MBF are shown in Fig 4 for global MBF values, and in Fig 5 for regional MBF values from apex_septal segment, respectively.

**Table 1:** Rest MBF (mL/min/g) regional and global values averaged for 12 healthy subjects, for two processing runs.

Segment	MBF1 (mL/min/g)	MBF2 (mL/min/g)	reproducibility (mL/min/g)	reproducibility%
SEPTUM	0.46	0.46	0.019	4.06
APEX_Sep	0.47	0.47	0.024	5.06
MID_Sep-I	0.49	0.49	0.030	6.03
MID_Sep-A	0.45	0.45	0.012	2.62
BASAL_Sep-I	0.45	0.46	0.025	5.54
BASAL_Sep-A	0.44	0.43	0.089	20.56
ANTERIOR	0.64	0.64	0.033	5.15
APEX_Ant	0.64	0.65	0.030	4.61
MID_Ant	0.63	0.63	0.034	5.42
BASAL_Ant	0.66	0.66	0.038	5.77
LATERAL	0.68	0.70	0.090	13.12
APEX_Lat	0.71	0.73	0.063	8.80
MID_Lat	0.67	0.70	0.191	27.91
BASAL_Lat	0.68	0.68	0.051	7.46
INFERIOR	0.79	0.80	0.057	7.24
APEX_Inf	0.81	0.83	0.115	14.00
MID_Inf-P	0.82	0.84	0.062	7.44
MID_Inf-I	0.90	0.91	0.053	5.85
BASAL_Inf-P	0.76	0.77	0.052	6.83
BASAL_Inf-I	0.70	0.71	0.047	6.64
GLOBAL	0.63	0.64	0.033	5.18

**Table 2:** Stress MBF(mL/min/g) regional and global values averaged for 12 healthy subjects, for two processing runs.

Segment	MBF1 (mL/min/g)	MBF2 (mL/min/g)	reproducibility (mL/min/g)	reproducibility%
SEPTUM	0.97	0.97	0.029	2.93
APEX_Sep	1.01	1.01	0.038	3.74
MID_Sep-I	1.15	1.15	0.035	3.05
MID_Sep-A	0.96	0.96	0.042	4.33
BASAL_Sep-I	0.98	0.98	0.046	4.65
BASAL_Sep-A	0.82	0.82	0.046	5.62
ANTERIOR	1.31	1.31	0.125	9.55
APEX_Ant	1.24	1.23	0.057	4.63
MID_Ant	1.30	1.29	0.093	7.20
BASAL_Ant	1.38	1.38	0.214	15.49
LATERAL	1.39	1.39	0.089	6.41
APEX_Lat	1.50	1.50	0.093	6.21
MID_Lat	1.33	1.33	0.085	6.35
BASAL_Lat	1.36	1.35	0.092	6.84
INFERIOR	1.92	1.91	0.087	4.54
APEX_Inf	2.15	2.15	0.114	5.32
MID_Inf-P	1.97	1.95	0.117	5.94
MID_Inf-I	1.88	1.85	0.188	10.08
BASAL_Inf-P	1.58	1.58	0.094	5.94
BASAL_Inf-I	1.72	1.72	0.107	6.20
GLOBAL	1.37	1.37	0.062	4.54

The resting global MBF values for the first and the second run were 0.63 ± 0.13 and 0.64 ± 0.13 mL/min/g, respectively, with a mean difference of -1% ± 2.6% (P = not statistically significant [NS]). The repeatability coefficient was 0.033 mL/min/g (5.18% of the mean). The pharmacological induced stress global MBF values were significantly higher, 1.37 ± 0.23 mL/min/g for the first run and 1.37 ± 0.24 mL/min/g for the second run, with a mean difference 0.1% ± 2.3% (P = NS) and a repeatability coefficient of 0.062 mL/min/g (4.54% of the mean).

The average per sector repeatability coefficients for rest MBF were 0.056 mL/min/g (8.5% of the mean) and stress MBF 0.089 mL/min/g (6.3% of the mean), which shows excellent repeatability for a majority of the segments. The range for the rest regional MBF values was from a very good mid_septum_A reproducibility of 0.012 mL/min/g (2.62% of the mean) to a marginal mid_lateral 0.191 mL/min/g (27.91% of the mean). The regional stress MBF values ranged from a very good mid_septum_I reproducibility of 0.035 mL/min/g (3.05% of the mean) to 0.214 mL/min/g (15.49% of the mean) basal_anterior reproducibility.

**Figure 6:**
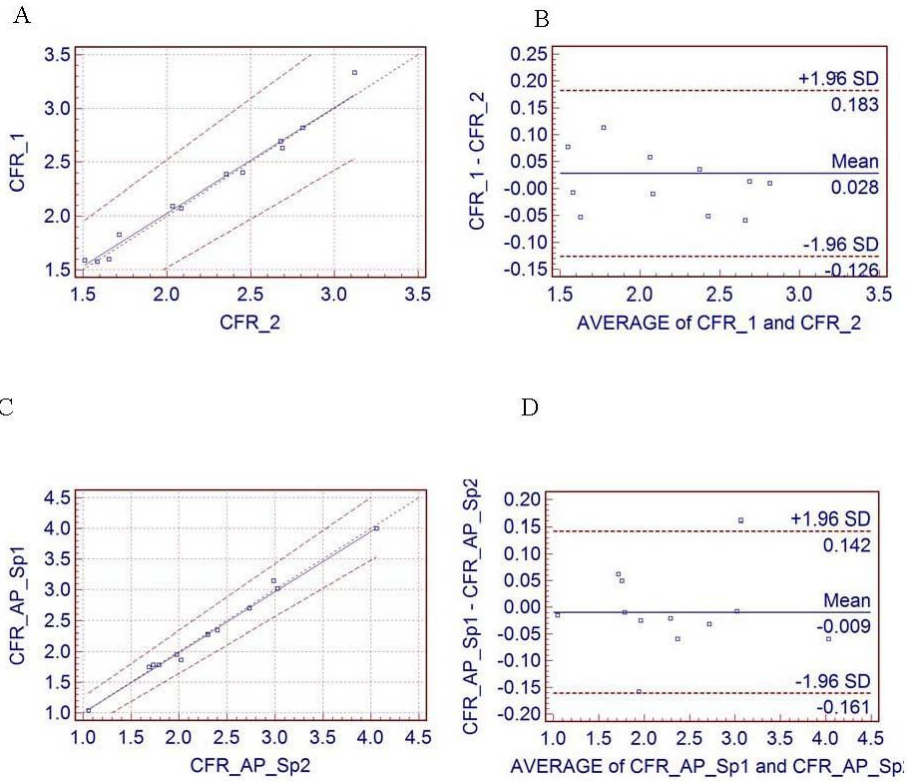
**CFR results**. (A) The Passing & Bablok regression scatter diagram with the regression line (solid line), the confidence interval for the regression line (dashed lines) and identity line (x = y, dotted line), for global CFR values (n = 12, r = 0.99) and (B) corresponding Altman-Bland plots. (C) The Passing & Bablok regression scatter diagram (n = 12, r = 0.99) for regional apex_septal CFR and (D) corresponding Altman-Bland plot for regional apex_septal CFR.

Table 3 gives the average of all 12 subjects, regional and global CFR with corresponding reproducibility and reproducibility expressed as percentage of the average value. Fig 6 shows the Passing & Bablok regression scatter diagram and the results of the Bland and Altman comparison between two runs for global and for regional apex_septal CFR, respectively.

**Table 3:** CFR regional and global values averaged for 12 healthy subjects, for two processing runs.

Segment	CFR1	CFR2	reproducibility	reproducibility%
SEPTUM	2.22	2.22	0.11	4.76
APEX_Sep	2.30	2.31	0.15	6.56
MID_Sep-I	2.56	2.59	0.25	9.51
MID_Sep-A	2.19	2.22	0.09	4.02
BASAL_Sep-I	2.33	2.29	0.19	8.25
BASAL_Sep-A	1.95	1.98	0.50	25.19
ANTERIOR	2.11	2.09	0.25	12.06
APEX_Ant	2.04	2.00	0.14	6.73
MID_Ant	2.11	2.08	0.22	10.66
BASAL_Ant	2.16	2.16	0.43	19.81
LATERAL	2.23	2.18	0.25	11.25
APEX_Lat	2.27	2.22	0.24	10.56
MID_Lat	2.13	2.07	0.30	14.16
BASAL_Lat	2.36	2.30	0.28	11.90
INFERIOR	2.70	2.66	0.19	7.10
APEX_Inf	2.83	2.80	0.25	8.86
MID_Inf-P	2.72	2.66	0.26	9.72
MID_Inf-I	2.53	2.49	0.23	8.98
BASAL_Inf-P	2.51	2.45	0.23	9.45
BASAL_Inf-I	3.15	3.12	0.39	12.43
GLOBAL	2.25	2.22	0.15	6.90

The first and second run global CFR values were 2.25 ± 0.56 and 2.22 ± 0.54, respectively, with a mean difference, 1.3% ± 14.3% (P = NS). The repeatability coefficient was 0.15 (6.9% of the mean).

The average per sector repeatability coefficient for CFR was 0.25 (10.6% of the mean) and it ranged from mid_septal_A regional reproducibility of 0.09 (4.02% of the mean) till basal_septal_A regional reproducibility of 0.50 (25.19% of the mean).

## Discussion

The advantages of ^13^NH_3_, ^15^O-labeled water (H_2_^15^O) over ^82^Rb for quantitative assessment of MBF are well known [19]. However, both of these tracers have notable disadvantages [20]. The most important is that the use of ^13^NH_3 _and ^15^O is restricted to sites with a cyclotron. In addition, ^15^O-labeled water is neither an approved tracer nor reimbursed for clinical imaging in the United States. The ability of ^15^O-labeled water to diffuse freely across plasma membranes makes this tracer a favourite for quantification of myocardial blood flow. However, this very property leads to poor contrast between the myocardium and cardiac blood pool. ^13^NH_3 _allows good quality gated and ungated images taking full advantage of the superior resolution of PET imaging. However, ^13^N ammonia images may be degraded by occasional intense liver activity, and increased lung activity in patients with lung congestion.

The main advantage of the ^82^Rb myocardium perfusion PET imaging is its availability without an expensive cyclotron.

Using the Daubechies wavelets for temporal smoothing of the dynamic data significantly improved MBF and CFR reproducibility. Without wavelet smoothing, the rest MBF values in both runs, although they were very similar (0.76 ± 0.61 vs. 0.76 ± 0.49 mL/min/g), had large standard deviations and global reproducibility was very marginal 0.23 mL/min/g (30% of the mean). The same held true for the stress global MBF values (1.75 ± 0.95 and 1.80 ± 1.06 mL/min/g) with poor reproducibility of 0.65 mL/min/g (36.6% of the mean).

Without wavelet smoothing, our results suggested that the regional reproducibility of MBF rest and stress values for many segments were even worse than global values and in general was not reproducible. As was shown before [9], the wavelet-based corrected ^82^Rb MBF values are lower than uncorrected MBF values. Our MBF values were reasonably close to those reported in the literature. For example, the recent ^82^Rb and ^13^NH_3 _rest MBF values [21] of 0.67 ± 0.13 mL/min/g and 0.69 ± 0.09 mL/min/g, were close to our corrected rest values (0.63 ± 0.13 and 0.64 ± 0.13 mL/min/g). However, our mean ^82^Rb corrected stress MBF values (1.37 ± 0.23 mL/min/g and 1.37 ± 0.24 mL/min/g) were slightly lower than those reported in the literature [22,23].

We believe that further improvement of assessing ^82^Rb rest and stress MBF values and CFR can be obtained by optimization of acquisition parameters, through additional comparisons with ^13^NH_3 _and ^15^O water measurements at sites with a cyclotron and using a larger population of subjects in comparisons. Also, separation of subjects by gender, age or disease, probably will make the assessment of ^82^Rb rest and stress MBF values more accurate. Recent results have also shown that factor analysis [24] can help in correcting input curves, providing better repeatability in the results.

The PMOD program itself can also be improved by a faster reading of data and by allowing creation of input TAC using the left atrium (LA) area in addition or instead of the LV cavity area. In some subjects with a small heart, a small LV cavity may not be the best choice for creating the input TAC, due to high cross talk from LV wall activity.

With these improvements, we believe that assessing ^82^Rb rest and stress MBF values and CFR, can be moved out from the research setting and applied more widely in the clinical environment.

## Conclusion

The results of the study demonstrate that processing of dynamic ^82^Rb images for PET assessment of MBF and CFR is repeatable. The rest and stress, global, as well as regional, MBF and CFR values were, in most cases, highly repeatable. The main advantage of the PMOD approach is that it automatically creates 16 cardiac segment MBF values, in addition to the septum, anterior, lateral, inferior and global value. Second, the PMOD has an option of wavelet temporal smoothing, which significantly improves the repeatability of MBF and CFR assessment.

## Competing interests

The authors declare that they have no competing interests.

## Authors' contributions

All authors have made substantial contributions in acquisition, processing and analysing of data, and writing the manuscript.

## Pre-publication history

The pre-publication history for this paper can be accessed here:



## References

[B1] Jadvar H, Strauss HW, Segall GM (1999). SPECT and PET in the Evaluation of Coronary Artery Disease. Radiographics.

[B2] Kaufmann PA, Camici PG (2005). Myocardial Blood Flow Measurement by PET: Technical Aspects and Clinical Applications. J Nucl Med.

[B3] Mullani NA, Goldstein RA, Gould KL (1983). Myocardial perfusion with rubidium-82. I. Measurement of extraction fraction and flow with external detectors. J Nucl Med.

[B4] Goldstein RA, Mullani NA, Marani SK, Fisher DJ, Gould KL, O'Brien HA (1983). Myocardial perfusion with rubidium-82. II. Effects of metabolic and pharmacologic interventions. J Nucl Med.

[B5] Goldstein RA, Mullani NA, Wong WH (1986). Positron imaging of myocardial infarction with rubidium-82. J Nucl Med.

[B6] Herrero P, Markham J, Shelton ME, Weinheimer CJ, Bergmann SR (1990). Noninvasive quantification of regional myocardial perfusion with rubidium-82 and positron emission tomography: exploration of a mathematical model. Circulation.

[B7] Herrero P, Markham J, Shelton ME, Bergmann SR (1992). Implementation and evaluation of a two-compartment model for quantification of myocardial perfusion with rubidium-82 and positron emission tomography. Circ Res.

[B8] Yoshida K, Mullani N, Gould KL (1996). Coronary flow and flow reserve by PET simplified for clinical applications using rubidium-82 or nitrogen-13-ammonia. J Nucl Med.

[B9] Lin JW, Robert R, Sciacca RR, Chou R-L, Laine AF, Bergmann SR (2001). Quantification of myocardial perfusion in human subjects using ^82^Rb and wavelet-based noise reduction. J Nucl Med.

[B10] Votaw JR, White M (2001). Comparison of 2D and 3D cardiac ^82^Rb PET Studies. J Nucl Med.

[B11] Knešaurek K, Machac J, Krynyckyi BR, Almeida OD (2003). Comparison of 2D and 3D ^82^Rb myocardial perfusion PET imaging. J Nucl Med.

[B12] Dimitrakopoulou-Strauss A, Strauss LG, Schwarzbach M, Burger C, Heichel T, Willeke F, Mechtersheimer G, Lehnert T (2001). Dynamic PET ^18^F-FDG Studies in Patients with Primary and Recurrent Soft-Tissue Sarcomas: Impact on Diagnosis and Correlation with Grading. J Nucl Med.

[B13] Wyss CA, Koepfli P, Mikolajczyk K, Burger C, Schulthess GK, Kaufmann PA (2003). Bicycle exercise stress in PET for assessment of coronary flow reserve: repeatability and comparison with adenosine stress. J Nucl Med.

[B14] Daubechies I (1992). Ten Lectures On Wavelets. SIAM.

[B15] Schiller NB, Shah PM, Crawford M (1989). Recommendations for quantification of the left ventricle by two-dimensional echocardiography. J Am Soc Echocardiogr.

[B16] Passing H, Bablok W (1983). A new biometrical procedure for testing the equality of measurements from two different analytical methods. Application of linear regression procedures for method comparison studies in Clinical Chemistry, Part I. J Clin Chem Clin Biochem.

[B17] Bland JM, Altman DG (1986). Statistical methods for assessing agreement between two methods of clinical measurement. Lancet.

[B18] (1976). Precision of Test Methods. I: Guide for the Determination and Reproducibility for a Standard Test Method.

[B19] Phelps EM (2004). PET – Molecular Imaging and Its Biological Applications.

[B20] Machac J (2004). Cardiac Positron Emission Tomography Imaging. Semin Nucl Med.

[B21] Lortie M, Mostert K, Kelly C (2005). Quantification of myocardial blood flow with 82Rb dynamic PET imaging. Eur J Nucl Med Mol Imagin.

[B22] Chareonthaitawee P, Kaufmann PA, Rimoldi O, Camici PG (2001). Heterogeneity of resting and hyperemic myocardial blood flow in healthy humans. Cardiovascular Research.

[B23] Nagamachi S, Czernin J, Kim AS, Sun KT, Bottcher M, Phelps ME, Schelbert HR (1996). Reproducibility of measurements of regional resting and hyperemic myocardial blood flow assessed with PET. J Nucl Med.

[B24] El Fakhri G, Sitek A, Kijewski MF, Di Carli MF, Moore SC (2005). Quantitative parametric ^82^Rb cardiac PET using generalized factor analysis of dynamic Sequences (GFADS) [abstract]. J Nuc Med.

